# A Protocol to Enhance INS1E and MIN6 Functionality—The Use of Theophylline

**DOI:** 10.3390/ijms17091532

**Published:** 2016-09-12

**Authors:** Milou Groot Nibbelink, Giulia Marchioli, Lorenzo Moroni, Marcel Karperien, Aart Van Apeldoorn

**Affiliations:** 1 Developmental BioEngineering, MIRA Institute of Biomedical Technology and Technical Medicine, University of Twente, Enschede 7522 NB, The Netherlands; g.marchioli@tue.nl (G.M.); h.b.j.karperien@utwente.nl (M.K.); a.a.vanapeldoorn@utwente.nl (A.V.A.); 2 Department of Complex Tissue Regeneration, MERLN Institute for Technology Inspired Regenerative Medicine, Maastricht University, Maastricht 6200 MD, The Netherlands; l.moroni@maastrichtuniversity.nl

**Keywords:** cell lines, insulin secretion in vitro, assay methods

## Abstract

In vitro research in the field of type I diabetes is frequently limited by the availability of a functional model for islets of Langerhans. This method shows that by the addition of theophylline to the glucose buffers, mouse insulinoma MIN6 and rat insulinoma INS1E pseudo-islets can serve as a model for islets of Langerhans for in vitro research. The effect of theophylline is dose- and cell line-dependent, resulting in a minimal stimulation index of five followed by a rapid return to baseline insulin secretion by reducing glucose concentrations after a first high glucose stimulation. This protocol solves issues concerning in vitro research for type I diabetes as donors and the availability of primary islets of Langerhans are limited. To avoid the limitations of using human donor material, cell lines represent a valid alternative. Many different β cell lines have been reported, but the lack of reproducible responsiveness to glucose stimulation remains a challenge.

## 1. Introduction

In vitro research in the field of type I diabetes is frequently limited by the availability of a functional model for islets of Langerhans. The development of a protocol for type I diabetes in vitro research is therefore highly desired. In vitro research is hampered as available cell lines do not have a similar responsiveness to glucose stimulation to islets. Cell lines are needed as the use of primary cells is limited due to donor shortages. Another common problem for the in vitro testing of human islets is represented by donor variability, which limits the comparison between different donors and different sets of experiments. These limitations suggest the need of a reliable and easily available method to produce pseudo-islets for in vitro research purposes. For this reason, many different cell lines have been created over the last decades [1]. Of all these β cell lines, mouse insulinoma MIN6 and rat insulinoma INS1E cell lines best reflect the physiological conditions, as both cell lines are responsive to glucose stimuli and they both express glucokinase [1]. Although β cell lines are commonly used, a major issue is the reproducible responsiveness to glucose stimulation. In particular, a low-high-low insulin secretion profile needs to be detected in response to low-high-low glucose stimulation, as seen in functional islets of Langerhans [2]. This is often not the case for both INS1E and MIN6 cells, as their insulin secretion does not return to the basal level after stimulation with low glucose for the second time. Therefore, either just the stimulation index or only the amount of insulin secreted in the first low glucose stimulation is shown in literature [3,4,5,6]. The stimulation index is a measure to express islet functionality. It is defined as the amount of insulin secreted under high glucose stimulation, divided by the basal insulin secreted in low glucose conditions. For islets of Langerhans, a threshold stimulation index of at least two defines a functional response, and often these cell lines do not reach this threshold level or display a reproducible behavior [7].

Ways to functionalize cells to secrete insulin upon glucose stimulation have been frequently explored in the field of fetal and neonatal islets. It is known that these cells hardly secrete insulin upon glucose stimulation [8,9,10]. Different components have been tested which act on different molecules/channels in the insulin secretion pathway, such as leucine, glipizide, theophylline, nicotinamide, and sodium butyrate [8,9,10,11]. Theophylline, a methylxanthine, has been described to enhance insulin secretion by stimulation of cAMP [8,9,11,12,13,14,15,16,17]. Theophylline inhibits phosphodiesterase activity, which leads to an increase in intracellular cAMP [8,13,16,17]. Adding theophylline to glucose buffers has already been applied in primary, fetal and neonatal islets as well as for administration in type I diabetes patients, in order to enhance their responsiveness to glucose stimulation [8,9,11,13,14]. However, until now the effect of theophylline has not been examined using INS1E and MIN6 cell lines.

The aim of this study was to develop a protocol to functionalize MIN6 and INS1E cell lines so they can be used as in vitro models for diabetes research. With the addition of theophylline, we show that MIN6 and INS1E aggregate cells become responsive to glucose stimulation in a reproducible manner and show stimulation indices >5 and a proper low-high-low insulin secretion profile. Additionally, we show a dose-dependent and cell line–dependent response. Since previous research has shown that insulin secretion is enhanced in pseudo-islets compared to cells in monolayers, our protocols were developed on MIN6 and INS1E pseudo-islets.

## 2. Results

### 2.1. The Effect of Theophylline on Insulin Secretion

**Figure 1:**
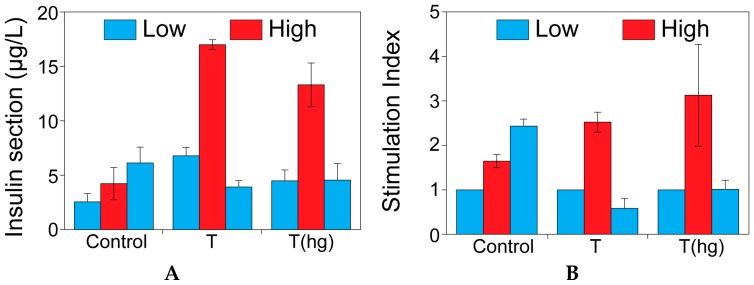
The effect of theophylline on insulin secretion. Insulin secretion (µg/L) (**A**) and stimulation index (**B**) of MIN6 cells cultured on tissue culture plastic (monolayer) without theophylline, with theophylline 10 mM in all the incubation buffers (T), and with theophylline 10 mM added only in the high glucose buffer (T(hg)).

Figure 1 shows the insulin secretion of MIN6 cells when stimulated with glucose. Cells were either stimulated with standard glucose buffers (1.67 and 16.7 mM) or with glucose buffers with 10 mM theophylline. Theophylline was added either in all buffers (T) or just in the high glucose buffer (T(hg)). Figure 1B shows the differences in stimulation indices between the different groups. Functionality is expressed as the stimulation index and it is defined as the amount of insulin secreted under high glucose stimulation, divided by the basal insulin secreted in low glucose conditions. The control group appeared to be non-functional whereas both theophylline groups exhibited functional insulin secretion profiles. Furthermore, no difference was seen in insulin secretion patterns between the two different theophylline groups. Although the low-high-low profile was visible when using theophylline compared to the control, only a marginal improvement in the stimulation index was seen. As these experiments were performed on monolayers, and the beneficial effect on function of cell aggregation has already been described in literature, further experiments were conducted with pseudo-islets [2,18,19].

### 2.2. The Effect of Theophylline on the Metabolic Activity of MIN6 and INS1E Cells

**Figure 2:**
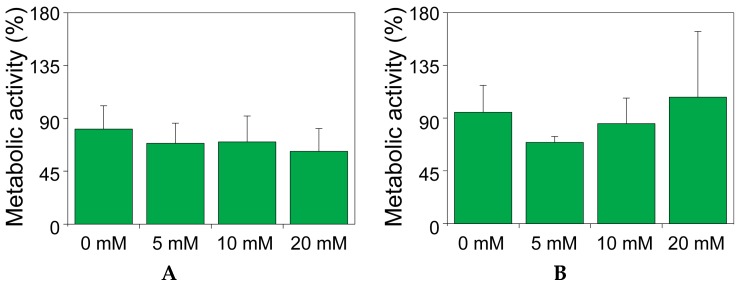
The effect of theophylline on the metabolic activity of MIN6 and INS1E cells. Metabolic activity of MIN6 (**A**) or INS1E (**B**) pseudo-islets cultured for 5 h in presence of different concentrations of theophylline. Data are presented as percentage of the metabolic activity at *t* = 0.

The effect on metabolic activity was assessed on both MIN6 (Figure 2A) and INS1E (Figure 2B) pseudo-islets for the duration of a glucose-induced insulin secretion test. Figure 2 shows the fold change of the metabolic activity after 5 h incubation compared to the initial basal activity. Again, no significant effect of theophylline was seen in both cell types and for none of the concentrations tested, confirming that theophylline exerted no toxic effect on MIN6 and INS1E pseudo-islets, for an incubation time of the duration of a standard function test.

### 2.3. Theophylline Concentration-Dependent Insulin Secretion of MIN6 and INS1E Pseudo-Islets

**Figure 3:**
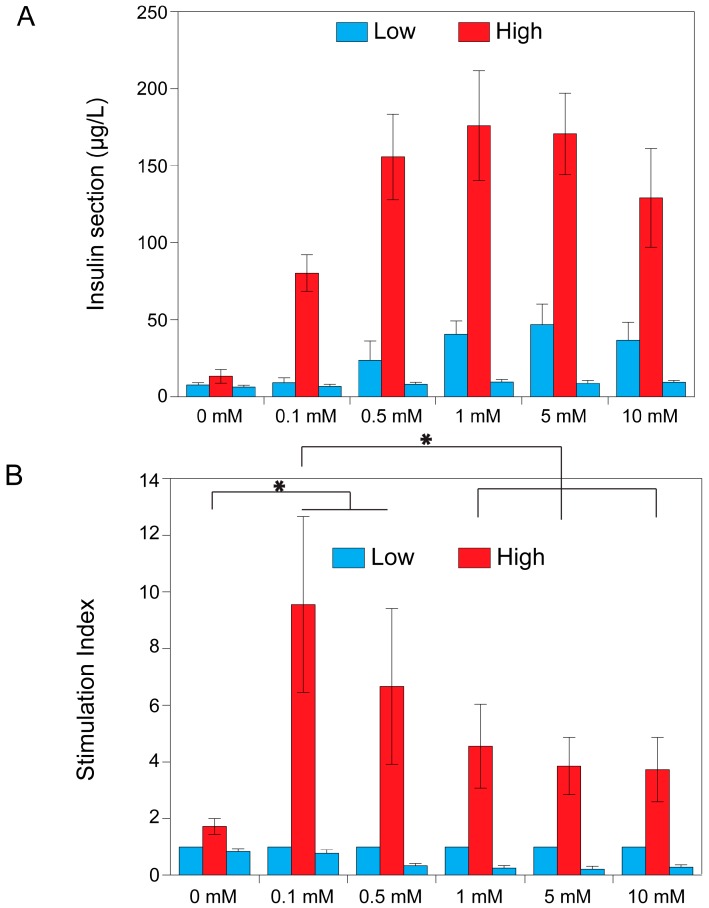
Theophylline concentration-dependent insulin secretion of MIN6 pseudo-islets. Dose-response results of the glucose-induced insulin secretion test on MIN6 pseudo-islets at different concentrations of theophylline. (**A**) Secreted amount of insulin (µg/L) is shown; in (**B**) data are normalized by the amount of insulin secreted in low glucose condition (stimulation index). Data are expressed as mean ± standard deviation and significant differences are indicated with * (*p* ≤ 0.05). Statistical analysis compares the stimulation indices in high glucose conditions.

**Figure 4:**
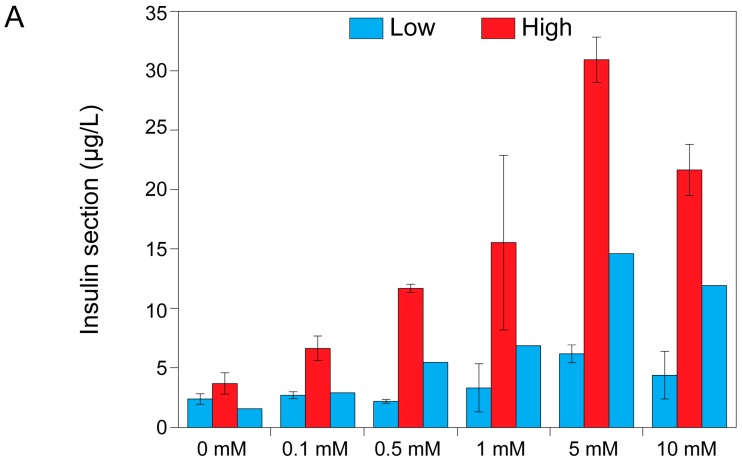

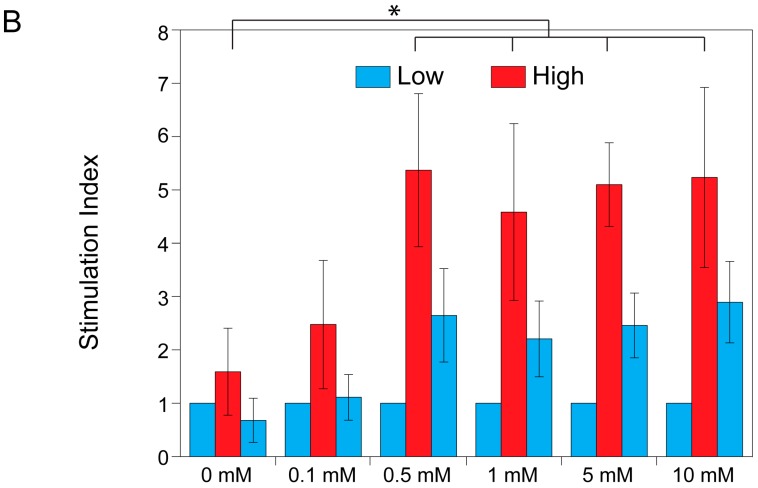
Theophylline concentration–dependent insulin secretion of INS1E pseudo-islets. Dose-response results of the glucose-induced insulin secretion test of INS1E pseudo-islets at different concentrations of theophylline. (**A**) Secreted amount of insulin (µg/L) is shown; (**B**) Data are normalized by the amount of insulin secreted in low glucose condition (stimulation index). Data are expressed as mean ± standard deviation and significant differences are indicated with * (*p* ≤ 0.05).

After establishing that theophylline did not impair viability and metabolic activity of both MIN6 and INS1E cells, the effect on insulin secretion was tested in MIN6 pseudo-islets (Figure 3) and INS1E pseudo-islets (Figure 4). Pseudo-islets were stimulated with different concentrations of theophylline to find the optimal concentration. Functionality is expressed as the stimulation index and it is defined as the amount of insulin secreted under high glucose stimulation, divided by the basal insulin secreted in low glucose conditions. Pseudo-islets were determined as functional when stimulation indices were above two, and a decrease in insulin secretion was seen when stimulated with low glucose buffer for the second time [7]. Figure 3 shows pseudo-islets’ functionality of MIN6 cells when stimulated with different concentrations of theophylline. Both the insulin secretion in μg/L (Figure 3A) and stimulation indices (Figure 3B) are depicted. The MIN6 pseudo-islets clearly showed a dose-dependent insulin secretion pattern. When looking at the insulin secretion (µg/L), the maximal effect appeared to be between 0.5 and 5 mM. However, when looking at the stimulation indices, the optimal concentration seems to be 0.1 mM. This was due to a lower insulin secretion when stimulated with low glucose for the 0.1 mM condition compared to the other concentrations of theophylline rather than a lower insulin production under high glucose stimulation, therefore resulting in a higher stimulation index. Despite the remarkable increase in insulin secretion by the addition of theophylline, insulin secretion returned to baseline after replacing the high glucose buffer with a low glucose buffer mimicking normal physiology.

Hilderink et al. showed an average stimulation index of 1.86 ± 0.7 for INS1E pseudo-islets [20]. This is comparable to our control group (Figure 4). Compared to the MIN6 pseudo-islets, INS1E pseudo-islets showed a similar insulin secretion pattern as MIN6 pseudo-islets. However, they seemed to be less sensitive to theophylline as they secreted less insulin when stimulated with 0.1 mM of theophylline compared to the MIN6 pseudo-islets (Figure 4A). Additionally, no detectable difference in the stimulation index was seen in the 0.5 to 10 mM range of theophylline concentration. Compared to MIN6 pseudo-islets, insulin secretion in INS1E pseudo-islets did not decrease as effectively to baseline when stimulated with the second low glucose buffer, particularly at the highest concentrations used.

## 3. Discussion

In this work, we demonstrated that the addition of theophylline to the glucose stimulation buffer is necessary to achieve a proper stimulation index both for MIN6 and INS1E pseudo-islets, thereby developing an in vitro protocol for diabetes research using cell lines instead of primary islets.

The generation of functional pseudo-islets for in vitro testing has been widely addressed in the literature and many authors report aggregation as a way to increase insulin secretion and functionality. Both islets and β cell line aggregates showed an increased glucose responsiveness in an aggregate configuration when compared to their dispersed counterpart [2,18,19,21,22]. In this work, we show that aggregation itself is not sufficient to generate a functional pseudo-islet, with a stimulation index comparable to native human tissue. As it was shown in Figure 3, the stimulation index in the control condition, without theophylline stimulation, is below two both for INS1E and MIN6 aggregates, which is considered the lowest threshold for defining an islet as functional [7]. Reproducible stimulation indices and a consistent return to the basal level of insulin secretion upon second stimulation with the low glucose condition is still a major issue. Many authors only publish first low and high stimulation, but neglect the importance of the return to the basal level, which is essential for defining an islet as functional [3,5,6]. In other papers only the amounts of insulin secreted in different conditions were compared among each other, but this does not give any indication about the proper functional response (low-high-low) of the tested cells [4].

With the addition of theophylline, we show that MIN6 and INS1E pseudo-islets are responsive with stimulation indices around five and a proper return to basal level in low glucose conditions. This return to baseline was more effective in MIN6 pseudo-islets than in INS1E pseudo-islets. Theophylline, a phosphodiesterase inhibitor, increases the intracellular cAMP levels by blocking its degradation and making it available longer for the increase of the cytosolic calcium concentration [23]. A mechanism proposed by Malaisse suggests that theophylline acts by mobilizing an intracellular source of calcium, originally located in the vacuolar system in the cytosol. In the absence of glucose, most of the calcium still escapes from the cytoplasm, but the simultaneous presence of glucose blocks this outward flux of calcium [23]. This mechanism explains why theophylline’s action is dependent on glucose stimulation and does not exert its effect in low glucose conditions, at least at low theophylline concentrations (0.1 mM) [23]. In fact, we show a dose-dependent response of MIN6 pseudo-islets to theophylline stimulation. In particular, for MIN6 pseudo-islets, the stimulation at a lower dose induced insulin secretion only in high glucose conditions and did not induce insulin release in basal medium (low glucose). On the contrary, higher theophylline concentrations stimulated insulin release also in low glucose conditions, thus lowering the overall stimulation index. For this reason, the action of theophylline on insulin secretion stimulation is glucose-dependent. Our results show that the addition of theophylline has no detrimental effect on the metabolic activity of INS1E and MIN6 pseudo-islets. This behavior was relatively different for INS1E pseudo-islets showing a lower sensitivity to theophylline action and highlighting a cell line–dependent response. More research would be necessary to understand the different behavior of MIN6 and INS1E pseudo-islets in depth: the difference could reside in species-specific characteristics or in a distinct fine-tuning regulation in the cAMP level in the insulin secretion mechanism. This difference might be explained by the higher glucokinase activity in MIN6 cells compared to INS1E cells. As shown by Arden and co-workers, the glucokinase activity in MIN6 cells is higher because of the higher insulin granule content in MIN6 cells [24]. It would be interesting to assess this cell line–dependent effect in other cell lines. Many different cell lines are available, even human β cell lines such as CM, TRM-1, Blox5 and the more recently developed EndoC-βH1 [1,25]. If glucokinase activity is indeed responsible for theophylline responsiveness, EndoC-βH1 might be a good candidate for theophylline [25]. However, the actual effect should be studied. Based on these results, we propose the use of 0.1 mM theophylline in the glucose-induced insulin secretion test of MIN6 pseudo-islets, since in these conditions the functional behavior of the islets of Langerhans is best resembled. In addition, we found that theophylline-stimulated MIN6 pseudo-islets showed a higher stimulation index and a better return to basal level upon second low glucose conditions. For INS1E pseudo-islets a higher theophylline concentration (5 mM) was necessary to significantly increase the stimulation index compared to the control (0 mM), but its subsequent return to basal level was sub-optimal. For in vitro purposes we therefore recommend the use of pseudo-islets of MIN6 cells.

## 4. Materials and Methods

### 4.1. Cell Culture

INS1E rat insulinoma cells (provided by Dr. Bruno Guigas, LUMC, Leiden, The Netherlands and Dr. Pierre Maechler, University Medical Center, Geneva, Switzerland) were cultured in RMPI (Gibco) with 2.05 mM Glutamax (Invitrogen, Bleiswijk, The Netherlands) supplemented with 5% (*v*/*v*) fetal bovine serum (FBS, Lonza, Verviers, Belgium), 100 U/mL penicillin and 100 mg/mL streptomycin (Gibco, Bleiswijk, The Netherlands), 10 mM 2-[4-(2-hydroxyethyl)piperazin-1-yl]ethanesulfonic acid (HEPES), 1 mM sodium pyruvate, and 50 µM freshly added β-mercaptoethanol (Gibco) at 37 °C and 5% CO_2_. Mouse insulinoma MIN6-B1 cells (provided by Dr. Phillipe Halban, University Medical Center, Geneva, Switzerland) were cultured in DMEM (Gibco) supplemented with 10% (*v*/*v*) FBS (Lonza), 100 U/mL penicillin and 100 mg/mL streptomycin, and 70 µM freshly added β-mercaptoethanol (Gibco) (37 °C, 5% CO_2_).

### 4.2. Agarose Microwell Fabrication and Controlled Pseudo-Islet Formation

For controlled pseudo-islet formation, cells were cultured in sterile agarose microwells. These agarose microwells were fabricated as described previously [26]. Polydimethylsiloxane (PDMS) negative molds containing micro pillars (200 µm) were sterilized (70% ethanol). A 3% agarose (UltraPureTM Gibco Invitrogen, Bleiswijk, The Netherlands) PBS solution was heated to 100 °C in a microwave (ESM117, ETNA, Duiven, The Netherlands). PDMS molds were placed inside six-well plate and filled with 6 mL agarose. Air bubbles were removed by centrifuging (300× *g*, 1 min). Solidification of the agarose was established at 4 °C (>30 min). Next, the molds were removed from the agarose using a sterile spatula. The agarose chips were punched out leaving a thin agarose wall on all sides to fit into a 12-well plate. Stable pseudo-islets were then created based on the work of Hilderink et al. [20]. MIN6 cells or INS1E cells were seeded onto the agarose chips (250 cells per aggregate). The plates were centrifuged (150× *g*, 1 min) and 1 mL of medium was carefully added to the chips. After 48 h at 37 °C pseudo-islets were flushed out of the chips.

### 4.3. Glucose Induced Insulin Secretion Test

A tailor-made Krebs buffer (115 mM NaCl, 5 mM KCl, 24 mM NaHCO_3_, Sigma, Diegem, Belgium) supplemented with 2.2 mM CaCl_2_, 20 mM HEPES (Gibco), 2 mg/mL bovine serum albumin, and 1 mM MgCl_2_ was prepared at pH 7.4 with different concentrations of theophylline (Sigma) (concentration range from 0.1 to 20 mM). Subsequently, the buffer was split into low (1.67 mM) and high glucose (16.7 mM). Cells were washed three times in low glucose buffer followed by a pre-incubation of 90 min in low glucose buffer. Cells were stimulated for 45 min in subsequent low, high and low glucose buffer with a three time wash step in low glucose between the high and second low. Samples were taken after each incubation, spun down (300× *g*, 3 min) and the supernatant was stored at −20 °C. Samples were analyzed using a rat insulin Elisa (Mercodia, Uppsala, Sweden) for INS1E samples and mouse insulin ELISA (Mercodia) for MIN6 samples.

### 4.4. The Effect of Theophylline on Insulin Secretion

MIN6 cells were seeded in a 12-well plate at a cell density of 30,000 cell/cm^2^. After 48 h insulin secretion upon glucose stimulation with the addition of theophylline (10 mM) in both low (1.67 mM) and high glucose (16.7 mM) Krebs buffers or only the high glucose (16.7 mM) Krebs buffer was tested. Insulin secretion was quantified by ELISA as described above.

### 4.5. The Effect of Theophylline on the Metabolic Activity of MIN6 and INS1E Cells

MIN6 and INS1E pseudo-islets were prepared as described above. Per condition approximately 950 pseudo-islets were seeded in a 96-transwell plate (40 μm, Millipore, Darmstadt, Germany). A Presto blue assay (Invitrogen) was performed following manufacturer’s protocol (1.5 h incubation) to determine their basal level of metabolic activity, before theophylline treatment. After the first presto blue, the pseudo-islets were washed three times in low glucose buffer with different theophylline concentrations (0, 5, 10, and 20 mM) and incubated for 5 h in the same low glucose buffer with added theophylline. After these 5 h of incubation, a second presto blue assay was performed (1.5 h incubation).

### 4.6. Theophylline Concentration-Dependent Insulin Secretion of MIN6 and INS1E Pseudo-Islets

MIN6 and INS1E pseudo-islets were prepared as described above. Per condition approximately 285 pseudo-islets were seeded in a 96-transwell plate (40 μm, Millipore). Six theophylline concentrations were tested (0, 0.1, 0.5, 1, 5, and 10 mM) by dissolving the theophylline in both low (1.67 mM) and high (16.7 mM) glucose Krebs buffers. Insulin secretion upon glucose stimulation was tested as described above.

### 4.7. Statistical Analysis

One-way ANOVA statistical analysis was performed followed by a Bonferroni post-hoc test. Data is expressed as mean ± standard deviation and significant differences are indicated with * (*p* ≤ 0.05). The analysis was performed using IBM SPSS statistic 20 software (IBM, Armonk, NY, USA).
